# Role of Enhanced Recovery After Surgery (ERAS) Protocols in Preventing Postoperative Acute Kidney Injury (AKI)

**DOI:** 10.7759/cureus.103782

**Published:** 2026-02-17

**Authors:** Zakariya Sattar, Aakif Yousaf, Muhammad Zubair, Muhammad R Pathan, Abdul Rasheed Zai, Javed Ahmed

**Affiliations:** 1 Otolaryngology, New Cross Hospital, The Royal Wolverhampton NHS Trust, Wolverhampton, GBR; 2 Surgery, Sahiwal Teaching Hospital, Sahiwal, PAK; 3 Medicine, Sahiwal Teaching Hospital, Sahiwal, PAK; 4 General Surgery, Indus Medical College, Tando Muhammad Khan, PAK

**Keywords:** aki stages, better outcomes, eras protocols, patient-centred care, postoperative blood loss

## Abstract

Background: Postoperative acute kidney injury (AKI) is a frequent and clinically consequential complication after major surgery.

Objective: To evaluate the association between enhanced recovery after surgery (ERAS) adherence and postoperative AKI incidence in patients undergoing major elective surgery.

Methodology: This prospective observational study was conducted at Sahiwal Teaching Hospital, Sahiwal, from November 2024 to April 2025. A total of 355 adult surgical patients were included; 182 (51.3%) received ERAS-based perioperative care, and 173 (48.7%) received conventional care. AKI was defined using the Kidney Disease: Improving Global Outcomes (KDIGO) criteria. Demographics, comorbidities, intraoperative variables, fluid balance, and ERAS adherence scores were recorded.

Results: AKI occurred in 46 patients (12.9%) overall. Incidence was significantly lower in the ERAS group compared with conventional care (15, 8.2%, vs. 32, 18.5%, *P *= 0.004). Postoperative length of stay was shorter with ERAS (6.2 ± 2.9 vs. 8.7 ± 3.4 days, *P *< 0.001), and ICU admission was less frequent (27, 14.8%, vs. 40, 23.1%, *P *= 0.04). On multivariable analysis, ERAS adherence ≥70% independently reduced the odds of AKI (aOR 0.46; 95% confidence interval (CI) 0.24-0.87; *P *= 0.015), whereas positive fluid balance >2 L (*P *= 0.001), baseline eGFR <60 (*P *= 0.021), and intraoperative hypotension (*P *= 0.020) were independent risk factors.

Conclusions: ERAS implementation is associated with a substantial reduction in postoperative AKI, shorter hospitalization, and fewer critical care escalations. Findings support positioning ERAS not only as a recovery pathway but as a renal-protective perioperative strategy.

## Introduction

Acute kidney injury (AKI) is not some benign lab abnormality we casually track; it’s a destabilizing clinical event that raises the risk of chronic kidney disease (CKD), cardiovascular complications, sepsis, prolonged hospitalization, dialysis dependence, and death. Even small, transient postoperative bumps in serum creatinine are now understood to reflect genuine structural and microvascular renal damage, with downstream morbidity that often becomes visible only at discharge [1]. Surgical patients, especially those undergoing major abdominal, cardiac, vascular, urologic, or oncologic procedures, face a perfect storm of renal insults: systemic inflammatory responses, ischemia-reperfusion injury, intraoperative hypotension, hemodilution, nephrotoxic exposures, hypovolemia, and the metabolic stress induced by fasting and surgical trauma [2]. With this level of pathophysiologic complexity, it’s naive to expect any single drug, biomarker, or monitoring device to *prevent AKI*. What’s needed is full-scale perioperative redesign. Enhanced recovery after surgery (ERAS) protocols represent exactly that: a coordinated, multidisciplinary, timeline-driven strategy that spans the preoperative, intraoperative, and postoperative phases to neutralize modifiable drivers of renal injury [3]. Unlike isolated anesthesia or nephrology consults that tend to be reactive, ERAS is built on anticipation rather than rescue [4]. It aligns anesthesia, surgery, nursing, nutrition, physiotherapy, pharmacy, and critical care into one unified clinical pathway that prevents complications before they materialize and addresses them early when they do. Mechanistically, ERAS disrupts renal injury at several physiologic checkpoints. A simple example is preoperative carbohydrate loading, which reduces insulin resistance, attenuates sympathetic overactivation, and promotes more stable renal perfusion, an upstream intervention with downstream protective effects [5].

Long-term starvation is avoided, and this avoids intravascular contraction, a primary cause of prerenal AKI. Goal-directed fluid therapy, during surgery, can prevent both hypoperfusion and fluid overload, which are two opposite and equally nephrotoxic conditions. Opioid-sparing analgesia decreases systemic vaso-dysregulation and inflammatory cascades that narcotics in high doses invoke. Postoperative feeding and mobilization in the early phase of the process attenuate the catabolic and inflammatory load, resulting in endothelial dysfunction and microvascular perturbation that precipitates tubular ischemia [6]. This biologic plausibility is supported by emerging clinical evidence. Several ERAS registries in colorectal, liver, and gynecologic surgery cohort studies have reported reduced postoperative renal dysfunction with an ERAS compliance level above 70-80, indicating that there may be a dose effect signal and not an incidental finding. Also, those hospitals that experience the most reduction of AKI according to ERAS are the ones that offer the best fluid balance and limit intraoperative hypotension, two adjustable pillars closely integrated into ERAS [7]. This is in line with KDIGO perioperative AKI prevention guidelines, which include precision in hemodynamics, avoidance of nephrotoxins, and mitigation of stress, which are inherent in ERAS without the need to use standalone protocols [8].

These positive indicators notwithstanding, significant uncertainties remain. ERAS programs vary greatly in terms of their composition, fidelity, and enforcement; that a particular institution has a program referred to as ERAS may have little to nothing in common with the program known as ERAS in a different institution [9]. Renal benefit is not a primary endpoint that is often defined in the trials of ERAS, so much of the existing inference remains retrospective and indirect. Moreover, it remains unclear whether all kidneys equally respond to ERAS or whether the benefit is confined to particular risk groups (e.g., diabetics, elderly, CKD stage 2-3, or high-bleed risk surgery). It is required to understand whether ERAS is generically renoprotective or whether ERAS is selectively renoprotective to transform ERAS into a generic versus organ-targeted organ-protective approach to recovery [10] [11].

The primary objective of this study was to evaluate the association between adherence to ERAS protocols and the incidence of postoperative AKI in patients undergoing major elective surgery. Secondary objectives included comparing postoperative length of hospital stay and ICU admission rates between patients managed under ERAS-based perioperative care and those receiving conventional care.

## Materials and methods

This prospective observational study was conducted at Sahiwal Teaching Hospital, Sahiwal, from November 2024 to April 2025. A total of 355 adult patients scheduled for elective gastrointestinal, hepatobiliary, gynecologic oncology, or urologic surgeries were enrolled. Patients following ERAS pathways constituted the exposure group, while those managed under traditional perioperative care constituted the comparison group. Allocation was naturalistic (according to attending team protocol preference), not randomized. Inclusion and exclusion criteria are given in Table 1.

**Table 1:** Inclusion and exclusion criteria.

Category	Criteria
Inclusion criteria	Age ≥ 18 years
	Elective major abdominal, oncologic, or pelvic surgery under general anesthesia
	Preoperative baseline serum creatinine available
	Expected postoperative length of stay ≥ 48 hours
Exclusion criteria	Preexisting end-stage renal disease or dialysis dependence
	Emergency surgery
	Known solitary kidney with baseline estimated glomerular filtration rate < 30 mL/min/1.73 m²
	Intraoperative conversion to an unplanned procedure
	Perioperative use of contrast within 24 hours before or after surgery

Data collection

Demographic variables, comorbidities (diabetes, hypertension, CKD stage), operative variables (type, duration, blood loss, episodes of intraoperative hypotension, vasoactive support), and postoperative laboratory values were recorded on a structured pro forma. The ERAS adherence checklist was completed prospectively. Patients in the ERAS arm received standardized components, including preoperative carbohydrate loading; avoidance of prolonged fasting; opioid-sparing multimodal analgesia; restrictive/goal-directed fluid management; normothermia maintenance; avoidance of nephrotoxic drugs; early oral nutrition; and early ambulation. Compliance was audited item-wise, and a composite adherence score (0-100%) was calculated. Patients managed under non-ERAS conventional perioperative pathways (NPO from midnight, liberal analgesic opioids, liberal crystalloids, delayed oral intake, and delayed mobilization) served as controls. The primary outcome was postoperative AKI defined by KDIGO criteria (increase in serum creatinine ≥ 0.3 mg/dL within 48 hours or ≥ 1.5× baseline within 7 days). Secondary outcomes were severity staging of AKI, need for renal replacement therapy, and postoperative length of stay. The ERAS protocol was implemented using a structured checklist encompassing preoperative carbohydrate loading and avoidance of prolonged fasting, intraoperative goal-directed fluid therapy with hemodynamic optimization and opioid-sparing analgesia, and postoperative early oral feeding and mobilization within 24 hours. Adherence to individual components was recorded prospectively, and a composite compliance score was calculated to quantify implementation fidelity.

Statistical analysis

Data were analyzed using SPSS version 26 (IBM Corp., Armonk, NY). Continuous variables were reported as mean ± standard deviation (SD) or median (interquartile range (IQR)) and categorical variables as *n* (%). Between-group comparisons used student’s t-test. Multivariable logistic regression was used to estimate adjusted odds ratios (aORs) for AKI with ERAS adherence as an exposure, controlling for age, baseline estimated glomerular filtration rate (eGFR), diabetes, operative time, fluid balance, and hypotension burden. A *P*-value < 0.05 was considered statistically significant.

## Results

A total of 355 patients were included, with 182 (51.3%) in the ERAS group and 173 (48.7%) in the conventional care group. Baseline factors were similar between groups. Men comprised 101 (55.5%) in ERAS versus 93 (53.8%) in conventional; diabetes was present in 68 (37.4%) versus 70 (40.5%); hypertension in 102 (56.0%) versus 99 (57.2%); baseline eGFR <60 mL/min/1.73 m² in 31 (17.0%) versus 29 (16.8%). Surgical distribution (GI/HPB/Gyn-Onco/Urology) in the ERAS group was 74 (40.7%)/41 (22.5%)/43 (23.6%)/24 (13.2%) and in the conventional group was 69 (39.9%)/38 (22.0%)/39 (22.5%)/27 (15.6%) (Table 2).

**Table 2:** Baseline demographic and clinical characteristics (N = 355). ERAS, enhanced recovery after surgery

Variable	ERAS (*n* = 182)	Conventional (*n* = 173)
Age (years), mean ± SD	57.1 ± 10.7	57.7 ± 11.1
Male sex, *n* (%)	101 (55.5%)	93 (53.8%)
Diabetes mellitus, *n* (%)	68 (37.4%)	70 (40.5%)
Hypertension, *n* (%)	102 (56.0%)	99 (57.2%)
Baseline eGFR <60 mL/min/1.73 m², *n* (%)	31 (17.0%)	29 (16.8%)
Type of surgery		
Gastrointestinal (GI), *n* (%)	74 (40.7%)	69 (39.9%)
Hepato-Pancreato-Biliary (HPB), *n* (%)	41 (22.5%)	38 (22.0%)
Gynecologic Oncology (Gyn-Onco), *n* (%)	43 (23.6%)	39 (22.5%)
Urology, *n* (%)	24 (13.2%)	27 (15.6%)

ERAS was associated with more favorable perioperative physiology. Intraoperative hypotension occurred in 39 (21.4%) versus 61 (35.3%) patients (*P *= 0.003). ICU admission was required in 27 (14.8%) versus 40 (23.1%) (*P *= 0.04). Postoperative length of stay was shorter in the ERAS group at 6.2 ± 2.9 days versus 8.7 ± 3.4 days (*P *< 0.001). Cumulative fluid balance at 48 hours was substantially lower in ERAS (+0.7 ± 0.5 L) compared to conventional care (+2.1 ± 0.8 L; *P *< 0.001) (Table 3).

**Table 3:** Intraoperative and early postoperative variables. ERAS, enhanced recovery after surgery; ICU, intensive care unit; SD, standard deviation; IQR, interquartile range

Outcome	ERAS (*n* = 182)	Conventional (*n* = 173)	*P*-value
Operative time (minutes), mean ± SD	191 ± 44	198 ± 47	0.18
Intraoperative hypotension episodes, *n* (%)	39 (21.4)	61 (35.3)	0.003
Blood loss (mL), median (IQR)	320 (240-480)	350 (260-520)	0.09
Cumulative fluid balance 48 hours (L), mean ± SD	+0.7 ± 0.5	+2.1 ± 0.8	<0.001
ICU admission, *n* (%)	27 (14.8)	40 (23.1)	0.04
Post-op LOS (days), mean ± SD	6.2 ± 2.9	8.7 ± 3.4	<0.001

The incidence of postoperative AKI was significantly lower with ERAS, seen in 15 (8.2%) versus 32 (18.5%) patients (*P *= 0.004). Stage 3 AKI occurred in 1 (0.5%) patient in the ERAS group compared with 4 (2.3%) in the conventional care group. Renal replacement therapy was required in 0 (0.0%) versus 3 (1.7%) patients, respectively. These differences indicate a clinically and statistically meaningful renal protective effect of ERAS (Table 4).

**Table 4:** Postoperative kidney outcomes. AKI, acute kidney injury; ERAS, enhanced recovery after surgery; KDIGO, Kidney Disease: Improving Global Outcomes

Variable	ERAS (*n* = 182)	Conventional (*n* = 173)	*P*-value
AKI (KDIGO) any stage, *n* (%)	15 (8.2)	32 (18.5)	0.004
Stage 1 AKI, *n* (%)	10 (5.5)	19 (11.0)	0.06
Stage 2 AKI, *n* (%)	4 (2.2)	9 (5.2)	0.14
Stage 3 AKI, *n* (%)	1 (0.5)	4 (2.3)	0.18
Renal replacement therapy, *n* (%)	0 (0.0)	3 (1.7)	0.08

Compliance with individual ERAS components was high: carbohydrate loading in 163 (89.6%), avoidance of prolonged fasting in 155 (85.2%), multimodal opioid-sparing analgesia in 171 (94.0%), goal-directed fluid therapy in 148 (81.3%), and nephrotoxin avoidance in 158 (86.8%). Composite adherence ≥70% was achieved in 137 (75.3%) patients, demonstrating consistent implementation of ERAS (Table 5).

**Table 5:** ERAS component adherence in ERAS cohort (n = 182). ERAS, enhanced recovery after surgery; NSAIDs, nonsteroidal anti-inflammatory drugs; ACEI, angiotensin-converting enzyme inhibitors

ERAS element	Achieved, *n* (%)
Preop carbohydrate load within 2-3 hours before anesthesia	163 (89.6)
Avoidance of prolonged fasting (>8 hours solids/>2 hours liquids)	155 (85.2)
Opioid-sparing multimodal analgesia	171 (94.0)
Goal-directed fluid therapy	148 (81.3)
Normothermia maintenance intra-op	165 (90.7)
Early oral feeding ≤24 hours post-op	142 (78.0)
Early mobilization ≤24 hours post-op	129 (70.9)
Nephrotoxin avoidance (NSAIDs/contrast/ACEI day 0-2)	158 (86.8)
Composite adherence ≥70%	137 (75.3)

High ERAS adherence (≥70%) independently reduced the odds of AKI (aOR 0.46; 95% CI 0.24-0.87; *P *= 0.015). Conversely, baseline eGFR <60 mL/min/1.73 m² (aOR 2.38; *P* = 0.021), intraoperative hypotension (aOR 2.11; *P *= 0.020), and fluid overload >2 L at 48 hours (aOR 2.94; *P *= 0.001) were strong independent predictors of AKI, underscoring the mechanistic relevance of hemodynamic and fluid optimization in ERAS (Table 6).

**Table 6:** Multivariable logistic regression for predictors of postoperative AKI (N = 355). AKI, acute kidney injury; eGFR, estimated glomerular filtration rate; ERAS, enhanced recovery after surgery; OR, odds ratio; CI, confidence interval

Predictor	Adjusted OR	95% CI	*P*-value
ERAS adherence ≥70%	0.46	0.24-0.87	0.015
Age ≥ 65 years	1.42	0.78-2.57	0.24
Diabetes mellitus	1.65	0.91-3.02	0.10
Baseline eGFR < 60	2.38	1.14-4.97	0.021
Intra-op hypotension	2.11	1.12-3.96	0.020
Fluid balance > +2 L in 48 hours	2.94	1.57-5.49	0.001
Operative time > 4 hours	1.36	0.71-2.61	0.35

## Discussion

Compliance with ERAS protocols, in this prospective cohort of 355 surgical patients, resulted in approximately 50 percent fewer cases of postoperative AKI than the traditional system of perioperative care. This protective influence remained despite statistical correction of classical risk factors of the kidney, like age, diabetes, pre-existing CKD, intraoperative hypotension, fluid overload, and duration of operation. These findings support the idea that ERAS is not simply a fast-track recovery intervention, but a biologically significant kidney-sparing intervention during perioperative care. There are a number of ERAS factors that are likely to have contributed to the observed renal effect [12]. To start with, metabolic homeostasis and intravascular stability (avoided by prolonged fasting and preoperative carbohydrate loading) lead to less prerenal insult. Second, intraoperative goal-oriented fluid therapy and improved hemodynamic management minimize the two extremes of the harm curve - ischemia caused by hypotension and interstitial pressure and venous congestion caused by fluid overload [13]. Third, opioid-sparing analgesia and early ambulation suppress the resulting neurohumoral response, leading to dysperfusion of renal tissue. Fourth, intentional prevention of perioperative nephrotoxins eradicated synergistic iatrogenic stimuli, especially in high-risk phenotypes. A combination of these mechanisms leads to an integrated response of hemodynamic, inflammatory, and endothelial injuries that are now known to be major contributors to postoperative AKI [14]. The degree of AKI change (8.2 vs. 18.5) is in line with data on ERAS registries of colorectal and urologic surgical units, where fluid stewardship and stress modulation progress has resulted in fewer renal complications. It is important to note that the mild creatinine bumps were not the only advantage in our group, as there were fewer stages of severity and dialysis needed in the ERAS group [15]. This same reduction in the number of ICU admissions and length of stay also attests to the systemic impact of ERAS on postoperative physiology other than that of the kidney. The causal argument is reinforced by the regression analysis, which shows that ERAS adherence was independent of protecting against the two strongest AKI drivers that were noticed: burden of hypotension and positive fluid balance. It is also worth noting that both of these mediators are directly altered by ERAS components, indicating that ERAS attains protection by weakening the effects of these mechanistic pathways instead of just choosing healthier patients [16].

Limitations

This study has several limitations. First, ERAS allocation was not randomized; patients were exposed based on treating team protocol preference, which introduces the possibility of selection bias and unmeasured confounding. Second, adherence to individual ERAS components varied and was measured as a composite score, making it impossible to identify which specific elements contributed most strongly to the observed renal protection. Third, AKI was defined using serum creatinine-based KDIGO criteria only; no biomarkers of subclinical kidney injury (e.g., NGAL or TIMP-2·IGFBP7) were assessed, and urine output-based staging was not reliably captured, potentially underestimating the true AKI burden. Fourth, the study was conducted at a single tertiary-care center, which may limit generalizability to lower-resource hospitals or different surgical populations. Fifth, postoperative follow-up was restricted to the in-hospital period; longer-term renal outcomes and the persistence or resolution of AKI beyond discharge were not evaluated.

## Conclusions

It is concluded that adherence to ERAS protocols significantly reduces the incidence of postoperative AKI in patients undergoing major elective surgery. The renal protection observed appears mechanistically consistent with the physiologic targets of ERAS stabilization of hemodynamics, restriction of unnecessary fluid load, attenuation of the surgical stress response, and avoidance of nephrotoxic exposures. Even after adjustment for established renal risk factors, ERAS compliance remained independently protective, underscoring its role not simply as a recovery-accelerator but as an organ-preserving perioperative strategy.
